# Association between maternal emotion socialization and emotion regulation in early adolescents with elevated internalizing symptoms: insights from multi-informant discrepancies

**DOI:** 10.3389/fpsyt.2025.1497007

**Published:** 2025-03-05

**Authors:** Junxuan Zhao, Elena Pozzi, Sylvia Chu Lin, Christiane E. Kehoe, Sophie S. Havighurst, Sarah Whittle

**Affiliations:** ^1^ Department of Psychiatry, The University of Melbourne, Melbourne, VIC, Australia; ^2^ Orygen, Melbourne, VIC, Australia; ^3^ Centre for Youth Mental Health, The University of Melbourne, Melbourne, VIC, Australia; ^4^ Mindful, Centre for Training and Research in Developmental Health, The University of Melbourne, Melbourne, VIC, Australia

**Keywords:** maternal emotion socialization, emotion regulation, informant discrepancy, multi-measurement, early adolescence

## Abstract

**Introduction:**

Although the relationship between parental emotion socialization and emotional competence, including emotion regulation, in children and adolescents has been extensively explored, there is a lack of research investigating this association in adolescents at high risk for mental health problems. The present study examined the association between maternal emotion socialization and emotion regulation in adolescents with high levels of internalizing symptoms, using multi-informant measurements (mother-reported, adolescent-reported, observer-reported). The study also explored whether discrepancies in the report of parental emotion socialization by different informants were related to adolescent emotion regulation, in addition to factors that may contribute to informant discrepancies.

**Methods:**

Participants were 70 female adolescents (mean age = 11.46 years, SD = 0.77) with high levels of internalizing symptoms and their mothers. Maternal emotion socialization (i.e., emotion dismissing and emotion coaching) was assessed using mother- and adolescent-reported questionnaires, and via observation during an emotion discussion task. Adolescent emotion regulation was reported by mothers and adolescents, while maternal emotion regulation was self-reported.

**Results:**

Adolescent-reported maternal emotion coaching and dismissing were significantly related to adolescent-reported adolescent emotion regulation. Informant discrepancies were not related to adolescent emotion regulation. Mothers higher in emotion regulation difficulties reported that their emotion coaching was more congruent with adolescent- and observer-reported emotion coaching, although this effect did not reach statistical significance.

**Discussion:**

Our findings highlight the value of adolescent-reported variables in parenting and adolescent emotion research. Additionally, mothers’ emotion regulation may influence their assessments of their emotion socialization behaviors.

## 1 Introduction

Emotion regulation (ER) is a key component of emotional competence (which comprises understanding of emotions, expression of emotions, and regulation of emotions). It is a complex construct, which refers to the ability to influence the generation, experience, and expression of emotion (1). The ability to monitor and evaluate emotions (e.g., be aware of and accept emotions) is also considered a part of ER (2). As an essential developmental skill, ER is closely linked to a young person’s well-being. Difficulties in ER are considered a broad risk factor for mental health problems during childhood and adolescence, including internalizing disorders (3). On the other hand, ER competence in young people is critical for attenuating the risk of developing internalizing disorders (4). Therefore, the development of ER is crucial for children and adolescents and investigating factors that may shape their ER is important for promoting healthy development and for identifying targets for prevention or early intervention.

The development of ER primarily occurs during childhood and adolescence (5). The family, as the primary environment for young people’s maturation, significantly influences the development of emotional competence (6). Among parenting factors, Eisenberg et al. (6) emphasized that emotion-related parenting has a significant impact on children’s development of emotional competence. They named emotion-related parenting philosophy and behavior ‘parental emotion socialization’, which refers to parents’ beliefs of emotions, how parents respond to children’s expression of emotion, discuss emotion with children, and express emotion themselves. Aligned with Eisenberg’s theory, Gottman et al. (7) proposed parents’ ‘meta-emotion philosophy’ (i.e., parents’ thoughts, beliefs and attitudes about their own and their children’s emotions) impacts their parenting in ways that can be described as either emotion coaching or emotion dismissing. Emotion coaching, a supportive form of parental emotion socialization, involves five key steps: (1) being aware of and understanding one’s and their children’s emotions, especially emotions of low intensity; (2) viewing children’s negative emotions as an opportunity to teach children how to manage such emotions; (3) being accepting of and empathic with children’s emotions; (4) helping children to describe their feelings; and (5) aiding them to solve problems and setting limits around behavior. In contrast, emotion dismissing, which represents an unsupportive form of parental emotion socialization, occurs when parents ignore, minimize or disapprove of children’s emotions. Both Gottman and Eisenberg hypothesized that parental emotion socialization directly affects children’s emotional competence, including ER.

Providing support for the aforementioned theoretical models, a number of studies have found associations between parent emotion socialization and children’s general emotional competence (e.g., 8, 9). There are fewer studies, however, that have examined links with ER, specifically. These studies have found that preschool children who receive more supportive maternal responses to their negative emotions use better ER strategies, such as context-appropriate ways to manage negative emotions (10). Unsupportive maternal emotion socialization has been found to be associated with children’s ER difficulties, including a lack of emotional awareness and inappropriate emotional expression (11). Unsupportive paternal emotion socialization has also been related to poorer children ER ability (12). While these studies explore the relationship between parental emotion socialization and ER, they have not investigated these associations in at-risk samples. Understanding these associations in those with greater mental health risks or difficulties can provide a comprehensive picture of emotion socialization in families and inform parenting interventions for children experiencing ER difficulties.

Additionally, while prior work has largely focused on younger children; there is little research examining how parental emotion socialization behavior relate to adolescents’ ER. Adolescence is a transition period between childhood and adulthood, when the brain’s cognitive control system, self-schema, and self-regulation capacity undergo significant changes (13). These emotional and behavioral changes make parent-adolescent relationships particularly challenging during this period, resulting in an increase in family conflicts and the establishment of more equal power dynamics between parents and children (14). Furthermore, female adolescents are more likely to experience emotional instability and internalizing symptoms compared to male adolescents (15), and maternal behavior has been found to more strongly correlated with ER capacity in female than male adolescents (16). Among the stages of adolescence, early adolescence is often marked by emotional instability due to heightened emotional reactivity and the rapid development of the brain (17). Consequently, early adolescence is a critical period to shape the development of ER. While some research has found supportive maternal emotion socialization to predict an improvement in the regulation of anger and sadness and lower levels of daily negative emotions in female adolescents (18, 19), further research is needed on the relationship between maternal emotion socialization behavior and early female adolescents’ general emotion regulation ability, rather than focusing solely on the regulation of specific emotions.

A further consideration in this research area concerns the measurement of parent emotion socialization and the correspondence of reports from different informants. More broadly, research findings in parenting studies often show inconsistencies when the same measures is administered to different informants (20, 21). A meta-analysis examining informant discrepancies in parenting found that there was a significant difference between parents’ and adolescents’ reports (22). Parents were more likely to perceive their parenting behavior as more positive compared to adolescents, who were more critical. Other work has found observer-reported parenting behavior to show weak associations with parent-reported parenting behavior (23). While coding observed behavior (e.g., during a parent-child interaction task) has been suggested as a more objective assessment of parental responses to their children’s emotions (24), it can be argued that observational measurements simply capture different aspects of parenting compared to self-report questionnaires, rather than one being more accurate than the other (25). Moreover, the type of task can influence the interaction between parents and children (26), so observational measures are dependent on the specific task and setting. Further, previous research has demonstrated that parenting behavior, as reported by different informants, has varying associations with child outcomes (27, 28). Therefore, employing a comprehensive approach that combines questionnaires with observational coding may enhance the validity of evaluating parental emotion socialization.

On the other hand, the discrepancy among informants might provide unique information beyond independent perspectives. Previous research suggests that a greater degree of informant discrepancy, specifically between parents and children, is associated with child maladjustment (29, 30). Discrepant reports might indicate that parents have a lack of awareness of their children’s development (30), which may be related to a lack of communication/insight or a high level of parent-child conflict. The discrepancy might also arise from parents’ psychopathology. Research has shown, for example, that higher levels of mental health problems in parents are related to lower congruence between parent- and adolescent-reported parenting (22, 31). De Los Reyes et al. (32) emphasized the importance of measuring both parents and children’s characteristics (e.g., maternal psychopathological symptoms) to better capture the dynamic processes underlying discrepant reports. Given that difficulties in ER are suggested to be a key transdiagnostic factor in both internalizing and externalizing disorders (33), and also that ER difficulties affect perception of affective behavior (34), ER difficulties might be the mechanism through which maladjustment (e.g., mental health disorders) leads to discrepancies in reporting parenting behavior. Considering factors that may increase discrepant reporting will help to better interpret research findings, and it may also be clinically relevant, for example, in relation to deciding intervention eligibility.

Although previous research has identified associations between informant discrepancies and child maladjustment (29, 30), there is no literature addressing the relationship between discrepancies in maternal emotion socialization and adolescent ER. Maternal emotion socialization, being a dyadic process, involves the subjective experiences of both mothers and children during interactions. Therefore, having insight into informant discrepancies and their relationship to adolescent emotional outcomes may offer a new viewpoint for exploring the potential influence of maternal emotion socialization on adolescents’ ER. In a clinical context, understanding these discrepancies will enhance the assessment of parenting behavior or family relationships for clinical intervention.

In summary, few studies have focused on the relationship between maternal emotion socialization and adolescent ER in high-risk sample (35). Further, existing research has primarily focused on community samples, rather than specifically targeting adolescents at risk for mental health problems associated with the experience of intense negative emotions. Additionally, previous studies have not addressed the relationship between informant discrepancies in maternal emotion socialization and adolescent ER.

The current study aimed to examine the relationship between maternal emotion socialization and ER in female adolescents with high levels of internalizing symptoms, which is a risk factor for developing internalizing disorders (36), using multi-informant measurements. Given evidence for sex differences in the effect of parental emotion socialization on adolescents’ emotional competences (6), and the higher prevalence of internalizing disorders in female adolescents compared to male adolescents (37), only female adolescents and their mothers were recruited to reduce heterogeneity and maximize statistical power. We further investigated the discrepancies between adolescent-reported, mother-reported, and observed maternal emotion socialization and their relationship to adolescent ER. Moreover, as mentioned above, difficulties in emotion regulation might be the mechanism leading to informant discrepancies in parental emotion socialization. To explore this further, we investigated whether adolescent or maternal emotion regulation difficulties contribute to any identified discrepancies. We hypothesized that (1) emotion coaching would be positively correlated and emotion dismissing would be negatively correlated with adolescent ER, regardless of the informant of parent emotion socialization and adolescent ER; (2) informant discrepancies in maternal emotion socialization would be associated with lower adolescent ER; (3) both adolescent and maternal difficulties in ER would contribute to the discrepancies in ratings for maternal emotion socialization; greater difficulties in both mothers and adolescents would predict greater informant discrepancy in maternal emotion socialization. This final hypothesis focuses on the causes of informant discrepancies by examining whether difficulties in ER at the individual level explain these discrepancies.

## 2 Materials and methods

### 2.1 Participants

Seventy female adolescents aged 10-12 years (mean age = 11.46 years, *SD* = 0.77) and their mothers (mean age = 41.89 years, *SD* = 11.62) were recruited from primary schools, clinical services, social media platforms, local universities, and flyers in local community centers. Mothers were predominantly Caucasian (81.4%), with a minority of mothers being Asian (11.4%), Mixed Heritage (4.3%), and Aboriginal or Torres Strait Islander (2.9%). The household types reported by mothers were as follows: 74.3% original family (both biological parents present), 17.1% sole parent family, 5.7% other, and 2.9% stepfamily (two parents, one being a step-parent to the adolescent in the study). Regarding education, the majority of mothers had completed or partially completed a university degree (88.6%), including a bachelor’s degree (34.3%), Honors degree (12.9%), or postgraduate degree (41.4%); 8.6% of mothers had completed a TAFE (Technical And Further Education)/Vocational training course, one mother had experienced primary school education, and one mother had received upper second school education. The majority of mothers reported their family’s gross annual income as more than $100,000 (above average income in Australia ( (38), 72.9%), the other mothers reported $60,000-$99,000 (15.7%), $40,000-$59,999 (8.6%), $20,000-$39,000 (1.4%), and 0-$19,999 (1.4%). Adolescents were considered eligible if they showed moderate to high levels of internalizing symptoms (e.g., anxiety and depression), with scores above the 50^th^ percentile on the self-reported Revised Children’s Anxiety and Depression Scale upon screening (39). Adolescents were excluded if they had a current diagnosis of a developmental or intellectual disorder, were currently using psychotropic medication, or had a history of head trauma.

### 2.2 Procedure

The current study utilized baseline data (pre-intervention) from a randomized controlled trial—Tuning in to Teens’ Brains (TINTB) study (ACTRN12621001304820). In the TINTB study, both mothers and adolescents were invited to a phone appointment where a study team member explained details of the study and conducted initial screening. Participants (both mothers and their adolescent daughter) provided verbal and written consent before participating in the study. Eligible participants were randomly assigned to either the intervention or the waitlist condition by a block-design computer randomizer. At the baseline assessment, mothers and their daughters completed questionnaires to assess maternal emotion socialization and adolescent ER. Mothers also reported on their own ER. Additionally, maternal emotion socialization was measured during an emotion discussion task performed in the families’ homes on Zoom with a researcher. Adolescents received compensation in the form of gift cards at a rate of $20 per hour. Mothers were not reimbursed for participation, but any reasonable travel and parking expense related to the study visit were covered in the form of parking/taxi vouchers.

### 2.3 Measures

#### 2.3.1 Maternal emotion socialization (mother and adolescent report)

The Emotions As a Child scale [EAC; (40)] is a 45-item questionnaire assessing maternal emotion socialization behavior related to adolescents’ anger, fear, and sadness (15 items for each emotion), with each item using a scale ranging from 1 (*not at all)* to 5 (*very strong*). The questionnaire includes five subscales: Reward, Override, Neglect, Magnify, and Punish. Among these subscales, Reward (example item: “When I was sad, my mother asked me what made me sad”) is categorized as a kind of supportive maternal emotion socialization (emotion coaching), and the sum of Neglect and Punish (example items include “When I was sad, my mother did not pay attention to my sadness” and “When I was sad, my mother told me to stop being sad”) are considered as a kind of unsupportive maternal emotion socialization (emotion dismissing). Seven items were reversed, higher scores on the Reward scales indicated greater supportive maternal emotion socialization; higher scores on the sum of Neglect and Punish scales indicated greater unsupportive maternal emotion socialization. Cronbach’s alpha was 0.93 for adolescent-reported emotion coaching, 0.89 for adolescent-reported emotion dismissing, 0.91 for mother-reported emotion coaching, and 0.87 for mother-reported emotion dismissing.

These measures of supportive and unsupportive maternal emotion socialization have been operationalized in the same way in previous research (41). The reason for excluding Override and Magnify is that the roles of these strategies in children’s development remain ambiguous. Override refers to suppressing a child’s emotional expression by diverting them or changing their emotion (40). Some have interpreted this distraction of emotion as minimizing children’s emotions, potentially negatively impacting children’s understanding of emotion (42). In O’Neal and Magai’s model (43), however, Override is categorized as a supportive parenting strategy. Furthermore, Magnify refers to when a child expresses an emotion, parents respond by expressing the same emotion with a stronger intensity towards the child. In O’Neal and Magai’s model (43), Magnify is classified as unsupportive maternal emotion socialization. Conversely, magnifying fear and sadness has been found to have a positive correlation with emotional warmth and empathy (44).

#### 2.3.2 Maternal emotion socialization (Observation)

The Emotion Discussion task [based on the work of Fivush et al. (45) and Suveg et al. (46)] is a 15-minute mother-adolescent interaction activity that asks the mother and adolescent to discuss occasions when the adolescent felt anxious, sad, and angry (5 minutes for each emotion). This task was performed at home with the mother-daughter dyad in the same physical room, and was recorded via Zoom (the one Zoom window captured both participants on video). A researcher, who was present throughout online, remained in the Zoom room but only provided cues to signal when to switch to the next emotion task. The observer kept their camera off and microphone muted during the task and unmuted only when it was time to move on to the next emotion task. The video recording was later coded for maternal emotion socialization.

For the coding of maternal emotion socialization behavior, a global rating system based on Baker et al.’s method (47) for coding emotion coaching and emotion dismissing was employed. Each subscale is rated on a scale from 1 (no signs of emotion coaching or emotion dismissing) to 5 (consistently showing emotion coaching or emotion dismissing during the task). Emotion coaching comprises five items: Structuring of emotion understanding, Sensitivity/acceptance, Validation and encouragement of emotional expression, Enthusiasm for discussion, and Intimacy/warmth/affection. For emotion dismissing, four items were coded: Derogation of child, Intrusiveness, Minimization of emotion/discouragement of expression, and Detachment/disinterest. Coders considered each subscale for emotion coaching and emotion dismissing to assign an overall 1 to 5 rating (“Overall Coaching”, “Overall Dismissing”). The overall scores for “Overall Coaching” and “Overall dismissing” were not calculated as a combination (e.g., sum or average) of the individual coding items. Instead, following Baker et al.’s method, coders viewed the recordings twice. During the first viewing, they generated an overall score to represent a global assessment of the parent’s behavior. During the second viewing, coders rated each item and compared these ratings to the overall coaching and dismissing scores. Structuring (for coaching) and Derogation of Child (for dismissing) were weighted most heavily when determining the overall scores. Coders considered any significant inconsistencies between item ratings and the overall score, making adjustments as necessary to ensure accuracy.

Two research students were first trained to deliver the Tuning into Teens intervention, a parenting intervention aimed at improving emotion coaching skills (48), and gain insight into the concept of parental emotion socialization. They were then trained to code the emotion discussion tasks by an experienced coder, utilizing data from the initial 5 recordings to familiarize themselves with the coding procedure, and then using 10 recordings to establish reliability. Inter-rater reliability was found to be acceptable, with a percent agreement of 92.86% (a <= one-point difference between raters was considered agreement; 49). During the inter-rater reliability check, coders aligned coding criteria and clarified any ambiguous description based on Baker et al.’s manual.

#### 2.3.3 Adolescent emotion regulation (mother and adolescent report)

The Emotion Expression Scale for Children [EESC; (50)] is an adolescent-reported 16-item questionnaire designed to assess adolescents’ ER. It measures two aspects of ER: emotion awareness and motivation to express emotion regulation. Participants rate each item on a scale from 1 (*not at all true*) to 5 (*extremely true*), with higher scores indicating greater difficulties in regulating emotions. The total score is recommended in use of measuring difficulties in ER (51). The sum of all item ratings was used to obtain a total score for emotion regulation difficulties (Cronbach’s alpha = .83).

The Difficulties in Emotion Regulation Scale – Parent Report [DERS-PR; (52)] was used to assess parent perceptions of adolescents’ ER. This 36-item mother-reported questionnaire encompasses six factors: Lack of emotional awareness, Lack of emotional clarity, Difficulty engaging in goal-directed behavior, Impulse control difficulties, Non-acceptance of emotional responses, and Limited access to emotion regulation strategies. Items are rated from 1 (*almost never*) to 5 (*almost always*), with higher scores indicating greater difficulties in emotion regulation. The sum of all items ratings was used (Cronbach’s alpha = .91).

#### 2.3.4 Maternal emotion regulation (mother report)

The Difficulties in Emotion Regulation Scale [DERS; (53)] is a 36-item questionnaire to assess ER through self-report. It has a similar structure to the DERS-PR, featuring the same six subscales. All item ratings were summed to obtain a total score, with higher scores indicating greater difficulties in ER (Cronbach’s alpha = .87).

#### 2.3.5 Supplementary analysis variables

##### 2.3.5.1 Demographic variables

Mothers’ race (1 = Aboriginal or Torres Strait Islander, 2 = Asian, 3 = Black people, 4 = Pasifika/Maori, 5 = White/Caucasian, 6 = Other/Mixed Heritage), family composition (1 = Original family (both biological parents present), 2 = Step family (two parents, one being a step parent to the child in the study), 3 = Sole parent family, 4 = Other), and family income were included as demographic variables.

##### 2.3.5.2 Mother internalizing symptoms (mother report)

The Depression Anxiety and Stress Scale [DASS; (54)] is a 21-item self-reported questionnaire to assess distress along three scales of depression, anxiety, and stress. Each item is rated from 0 (*Never*) to 3 (*Almost always*). The total scores for all items were used to represent mother internalizing symptoms (Cronbach’s alpha = .92).

##### 2.3.5.3 Adolescent internalizing symptom (adolescent report)

The Revised Child Anxiety and Depression Scale [RCADS; (39)] is a 47-item self-reported questionnaire designed to assess the level of internalizing symptoms in youth aged from 8 to 18 years. In the present study, Cronbach’s alpha = .91. Adolescent internalizing symptoms were used for screening and were not the primary focus of the present study. However, for descriptive purposes, correlations between internalizing symptoms and other variables are presented.

##### 2.3.5.4 Sexual maturity status (mother report)

The Sexual Maturity Scale Parent-Report [SMS-PR; (55)] is a set of line drawings of girls bodies, based on five Tanner stages of pubertal development (56). Parents were presented with images of breast and female pubic hair development and asked to pick the image that most represents their adolescent’s development. If ratings of pubic hair and breast development were different, the higher value was used (57).

### 2.4 Statistical analysis

All analyses were conducted in RStudio Version 2023.6.1 (58). One participant’s recording of the emotion discussion task was missing, and three participants did not complete the emotion discussion task due to personal reasons. Multiple imputation of these missing data was run with the mice package in RStudio (v3.16.0; 59).

The first hypothesis (i.e., the relationship between maternal emotion socialization and adolescent ER) was examined using bivariate correlations between maternal emotion socialization reported by multi-informants (mothers, adolescents, and observers), adolescents’ self-reported and mother-reported ER, self-reported maternal ER, and self-reported and mother-reported adolescent internalizing symptoms. To test the second hypothesis (i.e., investigate the relationship between informant discrepancy and adolescent ER), we used polynomial regression analyses to investigate informant discrepancy (60). The direct difference score correlation indicates (a) differences in correlations between reports from two different informants and the second variable, and (b) differences in the variances of two informant reports (60, 61). The use of difference scores has been extensively critiqued. Difference scores cannot be produced when two informants’ reports have equal variance and equal correlations with the outcome, even if the difference score is significantly correlated with the second variable. Polynomial regression analyses can be used as an alternative to difference scores to investigate informant discrepancy (60). Interaction terms added in the polynomial regression model directly assess whether high (or low) scores from one informant show a stronger or weaker association with the outcome when scores from the other informant are also high (or low). Polynomial regression analyses were thus employed to test the impact of interactions between informant reports on adolescent ER. In the current study, separate models were run for each pair of informants and for emotion coaching and emotion dismissing. If the interaction terms in polynomial regression models were associated with the outcome, we used *post hoc* tests to identify the pattern of associations (30). Linear regression models were used to examine the role of mothers’ and adolescents’ ER in informant discrepancies. First, models were tested for the moderating role of adolescent ER in the association between maternal, self-rated- and observer-reported emotion socialization. Separate models were tested for emotion coaching and emotion dismissing. Then, models were tested for the moderating role of maternal ER in the association between adolescent and observer-reported maternal emotion socialization; separate models were tested for emotion coaching and emotion dismissing. All variables were mean-centered before running the polynomial and linear regression models (62).

Finally, supplemental analyses repeated primary analyses with the inclusion of covariates. Demographic variables were included as covariates if they were significantly associated with study variables. Puberty status was also included as a covariate. In addition to emotion regulation difficulties, parents’ and adolescents’ characteristics (e.g., internalizing symptoms and demographic variables) may also explain discrepancies in reporting parenting (22). Therefore, internalizing symptoms and demographic variables were tested to determine whether they predicted informant discrepancies and contributed to these discrepancies in the supplemental analyses.

## 3 Results

### 3.1 Descriptive data

**Figure 1:**
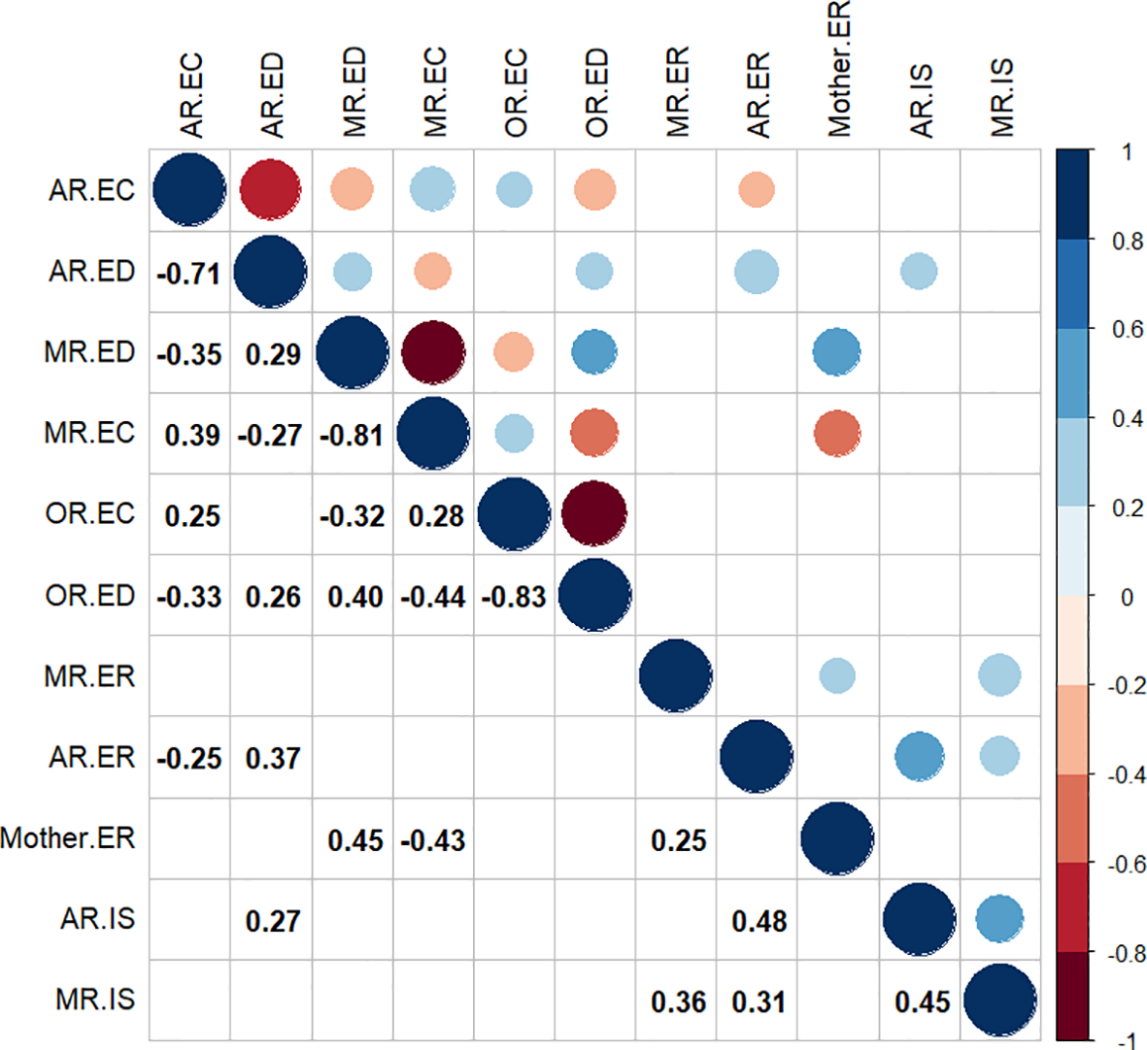
Bivariate Correlations. Non-significant coefficients are left blank in this figure. AR, adolescent-reported; MR, mother-reported; OR, observer-reported; EC, emotion coaching; ED, emotion dismissing; ER, emotion regulation; Mother. ER, self-reported maternal emotion regulation; IS, internalizing symptoms.

**Figure 2:**
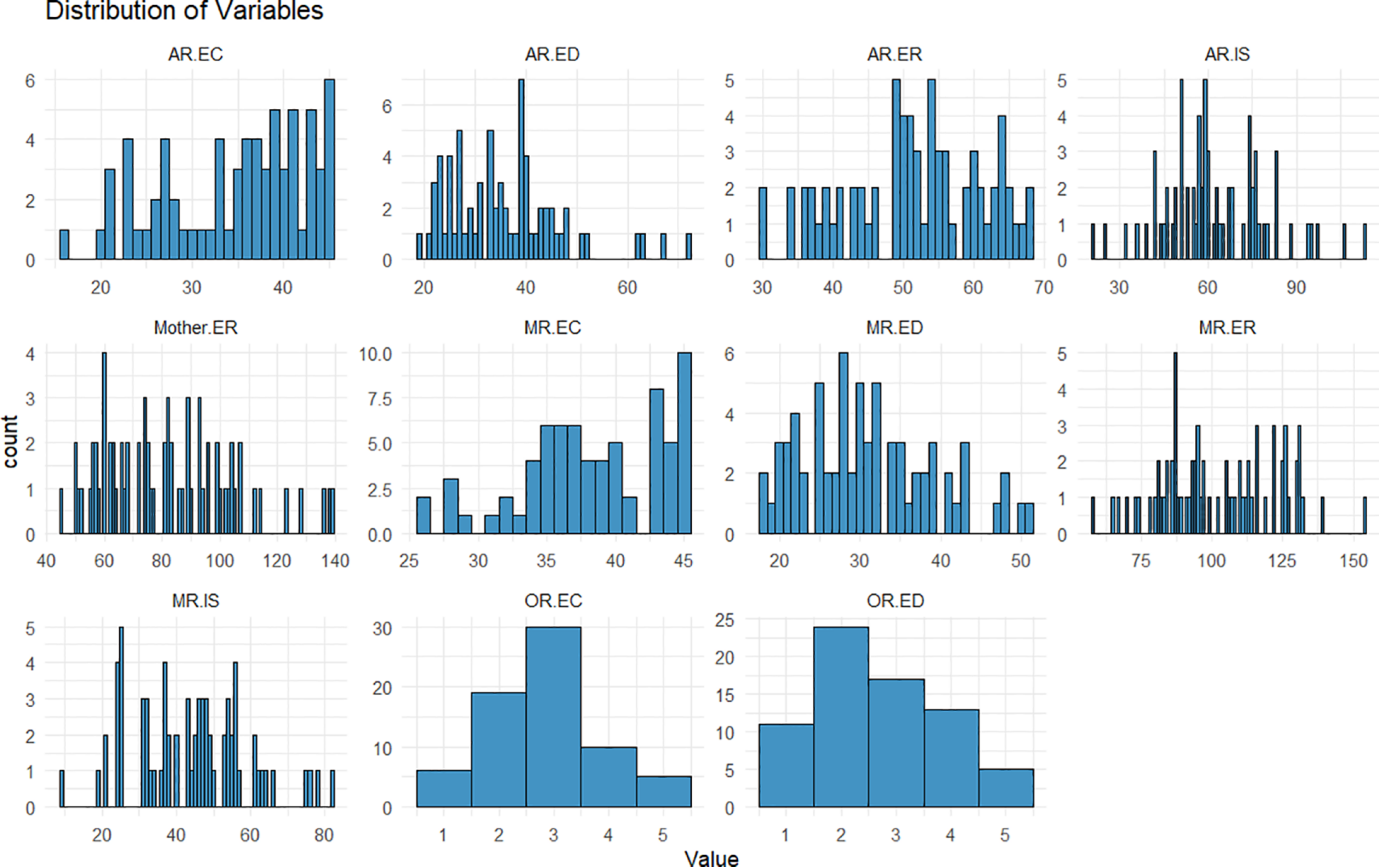
Distribution plots for maternal emotion socialization, adolescent emotion regulation, mother emotion regulation, and internalizing symptoms. AR, adolescent-reported; MR, mother-reported; OR, observer-reported; EC, emotion coaching; ED, emotion dismissing; ER, emotion regulation; Mother.ER, self-reported maternal emotion regulation; IS, internalizing symptoms.

Means and standard deviations (*SD*s) for emotion coaching, emotion dismissing, and mothers’ and adolescents’ ER, and demographic variables as reported by multi-informants, are presented in **Table 1**. Bivariate correlations between variables are presented in **Figure 1** (non-significant correlations are presented in **Supplementary Table S1**). The distribution of adolescent-reported maternal emotion dismissing was highly skewed (skewness = 1.04), and the distributions of other variables were normal or moderately skewed (range = -0.52 to 0.63), shown in **Figure 2**. Mothers rated their emotion coaching skills higher than adolescents, *t*(69) = -3.95, *p* <.001; mother reported emotion dismissing was significantly lower than that reported by adolescents, *t*(69) = 3.40, *p* <.005. The current study utilized different questionnaires for mother and adolescent-reported ER. Therefore, a t-test was not conducted to measure the informant discrepancy in adolescent ER. Similarly, as observer-reported maternal emotion socialization used a different scale compared to adolescent and mother report, we did not test for informant discrepancies.

**Table 1:** Descriptive statistics.

Variable	*M*	*SD*	Range
Emotion Coaching (Adol-report; EAC)	34.77	7.78	16-45
Emotion Coaching (Mother-report; EAC)	38.30	5.20	26-45
Emotion Coaching (Observation)	2.84	1.02	1-5
Emotion Dismissing (Adol-reported; EAC)	35.90	11.08	19-72
Emotion Dismissing (Mother-reported; EAC)	31.13	8.29	18-51
Emotion Dismissing (Observation)	2.67	1.16	1-5
Emotion regulation (Adol-report; EESC)	51.49	9.87	30-68
Emotion regulation (Mother-report; DERS-PR)	102.24	20.37	58-154
Maternal Emotion regulation (Mother-report; DERS)	81.91	22.62	45-139

Adol, adolescent; DERS, The Difficulties in Emotion Regulation Scale; DERS-PR, The Difficulties in Emotion Regulation Scale-Parent Report; EAC, The Emotions As a Child scale; EESC, The Emotion Expression Scale for Children; *M*, Mean; *SD*, Standard Deviation.

### 3.2 Associations between maternal emotion socialization, adolescent emotion regulation, and maternal emotion regulation from multi-informants

Adolescent-reported maternal emotion socialization and adolescent ER showed a correlation with a medium effect size (Cohen’s *d* = 0.53, *p* <.05 for emotion coaching, Cohen’s *d* = 0.8, *p* <.005 for emotion dismissing; see **Figure 1**). This correlation was positive for emotion coaching and negative for emotion dismissing. Neither maternal- nor observer-reported maternal emotion socialization was significantly correlated with adolescent ER. Additionally, adolescent-reported internalizing symptoms showed a medium effect size correlation with adolescent-reported emotion dismissing (Cohen’s *d* = 0.56, *p* <.05) and a large effect size correlation with adolescent-reported ER (Cohen’s *d* = 1.09, *p* <.001). Mother-reported internalizing symptoms were not correlated with maternal emotion socialization but were correlated with both adolescent- and mother-reported adolescent ER, with medium to large effect sizes (Cohen’s *d* = 0.65, *p* <.05 and Cohen’s *d* = 0.77, *p* <.005).

### 3.3 Informant discrepancies in maternal emotion socialization as predictors of adolescent emotion regulation

The second hypothesis tested informant discrepancies in maternal emotion socialization as predictors of adolescent ER. None of the interaction terms involving adolescent report (adolescent-reported X mother-reported emotion socialization, *p* = .136 for emotion coaching and *p* = .752 for emotion dismissing; adolescent-reported X observer-reported emotion socialization, *p* = .377 for emotion coaching and *p* = .609 for emotion dismissing) reached statistical significance, indicating that the discrepancies between adolescent and other reports of emotion socialization were not associated with adolescent-reported adolescent ER. Similarly, none of the interaction terms involving mother report (mother-reported X adolescent-reported emotion socialization, *p* = .803 for emotion coaching and *p* = .584 for emotion dismissing; mother-reported X observer-reported emotion socialization, *p* = .379 for emotion coaching and *p* = .175 for emotion dismissing) reached statistical significance, indicating that the discrepancies between mother and other reports of emotion socialization were not associated with mother-reported adolescent ER. Details of results are presented in the Supplementary Material (**Supplementary Tables S2–S5**). Therefore, no additional tests to probe interaction patterns were conducted.

### 3.4 Maternal and adolescent emotion regulation and informant discrepancies

Our third hypothesis was in relation to the contribution of both adolescent and maternal difficulties in ER to the informant discrepancy for emotion socialization. Linear regression models revealed that neither adolescent nor maternal difficulties in ER significantly moderated the associations between maternal emotion socialization reported by different informants (**Table 2**). However, the interaction between adolescent-reported emotion coaching and maternal difficulties in ER was approaching significance in predicting mother-reported emotion coaching (*β* = .01*, SE* = .003*, p* = .053).

**Table 2:** Linear regression testing the contribution of adolescent or maternal emotion regulation to informant discrepancies.

Dependent variable: Adolescent-reported emotion coaching				
	*β*	*SE*	*p*	R^2^
Adolescent ER	-0.21	0.09	.017*	.26
Mother-reported emotion coaching	0.61	0.17	.001***	
Observed emotion coaching	0.76	0.87	.384	
Mother-reported x Adolescent ER	0.02	0.02	.331	
Observed x Adolescent ER	0.09	0.09	.303	
Dependent variable: Adolescent-reported emotion dismissing				
	*β*	*SE*	*p*	R^2^
Adolescent ER	0.41	0.12	.002**	.24
Mother-reported emotion dismissing	0.34	0.16	.040*	
Observed emotion dismissing	1.04	1.15	.371	
Mother-reported x Adolescent ER	0.01	0.02	.492	
Observed x Adolescent ER	-0.02	0.13	.852	
Dependent variable: Mother-reported emotion coaching				
	*β*	*SE*	*p*	R^2^
Mother ER	-0.08	0.02	.002**	.35
Adolescent-reported emotion coaching	0.19	0.07	.001**	
Observed emotion coaching	0.77	0.55	.163	
Adolescent reported x Mother ER	0.01	0.003	.053	
Observed x Mother ER	-0.01	0.02	.748	
Dependent variable: Mother-reported emotion dismissing				
	*β*	*SE*	*p*	R^2^
Mother ER	0.12	0.04	.002**	.36
Adolescent-reported emotion dismissing	0.11	0.08	.148	
Observed emotion dismissing	1.80	0.76	.021*	
Adolescent- reported x Mother ER	-0.01	0.01	.111	
Observed x Mother ER	0.05	0.03	.107	

*p<0.05, **p<0.01, ***p<0.001. ER, emotion regulation; *SE*, standard error.

**Figure 3:**
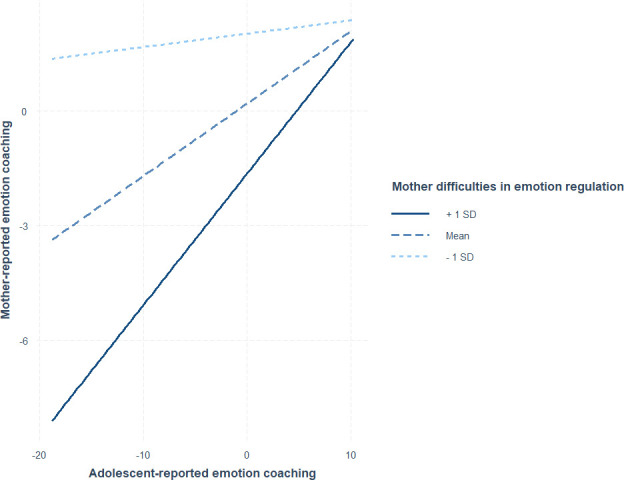
The association between mother- and adolescent-reported emotion coaching at high and low levels of maternal difficulties in emotion regulation.

The trend finding indicates that when mothers reported greater difficulties in ER, there was a positive association between mother-reported emotion coaching and adolescent-reported emotion coaching (see **Figure 3**). This finding thus shows when mothers reported greater difficulties in ER, there was greater concordance between mother-reported and adolescent-reported emotion coaching.

### 3.5 Supplementary analysis

Results of the supplementary analysis including race of the mother, family composition, family income, pubertal maturity status, and maternal internalizing symptoms as covariates are available in the **Supplementary Materials**. When primary analyses were rerun including these covariates, results were unchanged. The supplementary analysis examining whether adolescent and maternal internalizing symptoms, family composition, and mother’s race predicted informant discrepancies is presented in the **Supplementary Materials (S6–S17)**. The supplementary analysis showed informant discrepancies in maternal emotion socialization were not associated with supplementary variables (adolescent and maternal internalizing symptoms, family composition, and mother’s race); and all these supplementary variables did not significantly contribute to informant discrepancies in maternal emotion socialization.

## 4 Discussion

In studying the relationship between maternal emotion socialization and ER in early female adolescents using multi-informant measurements (mother-reported, adolescent-reported, and observational report), there were three key findings: (1) only adolescent-reported, but neither mother-reported nor observational reports, of maternal emotion coaching and emotion dismissing were associated with adolescent ER; (2) informant discrepancy was not related to adolescent ER; and (3) maternal ER was found to moderate the association between mother- and adolescent-reported emotion coaching at trend level only. Although the results are preliminary due to the sample size, and some only reached a trend level of significance, we will discuss them in light of the novel nature of the study. However, we acknowledge that these findings should be interpreted with caution, and they require further validation.

Correlational analyses suggested that only adolescent-reported (but not mother- or observer-rated) emotion coaching and emotion dismissing were negatively and positively (respectively) related to adolescent ER; and adolescent-reported internalizing symptoms was positively related to adolescent-reported emotion dismissing. This finding partially supports the theoretical model of parental emotion socialization that supportive maternal emotion socialization helps children understand and regulate emotions, while unsupportive maternal emotion socialization is associated with children’s low emotional competence (6, 7, 63). Regarding internalizing symptoms, our findings aligns with previous meta-analysis study, which showed that parental warmth has a small association with internalizing symptoms, whereas neglectful parenting is strongly associated with high levels of internalizing symptoms (64). This suggests that supportive and unsupportive parental emotion socialization may function differently in adolescents’ development.

Different informant reports yielded different results, consistent with prior research (21). The finding of adolescent-reported emotion socialization being associated with adolescent ER is consistent with some prior research on male and female adolescents (65). Research has shown, for example, that adolescents’ perceptions of parenting behavior have stronger associations with their ER than what is reported by parents (66, 67). One study with adolescent males and females showed that mothers’ reports of adolescents’ behaviors, mental health symptoms, and family environment do not predict adolescents’ emotional functioning (68).

However, that mother- and observer-reported emotion socialization were not associated with adolescent ER is inconsistent with other studies. Previous findings, in both female and mixed-sex samples, demonstrated that parent- and observer-reported supportive maternal emotion socialization was associated with better child emotional competence (8–10, 19). However, previous studies included community samples, whereas participants in the current study consisted of early female adolescents with high levels of internalizing symptoms. It is possible that because at-risk female adolescents are more likely to experience intense negative emotions (69), this might influence the impact of maternal emotion socialization (6). ER is largely an internal process; parents can only infer, but not directly observe, their children’s internal emotion states and thoughts (70). Therefore, adolescent-reported ER may offer more insight into adolescent subjective emotional experience compared to others’ reports.

Previous research focused on the effect of informant discrepancies on children’s behavior and mental health, and general parenting behavior (22, 29, 30). To our knowledge, the current study is the first to empirically investigate informant discrepancies in maternal emotion socialization. Consistent with previous research (22), mothers perceived their emotion-related parenting behavior to be more positive than their adolescent daughters’ ratings, such that they reported significantly higher scores for emotion coaching and significantly lower scores for emotion dismissing. The underlying reason for the discrepancy between mothers’ and adolescents’ reports could be due to several reasons. First, there could be a social desirability bias where parents want to present themselves more positively than the reality (71). Second, the informant discrepancy could be interpreted through the maladaptive hypothesis. This hypothesis suggests that the informant discrepancy is maladaptive for adolescent development because it may reflect problems in family relationships; there may be a lack of communication between parents and adolescents, resulting in adolescents perceiving parenting behavior more negatively (72). Conversely, the informant discrepancy could be interpreted as reflecting different agendas during the transition from childhood to adolescence (73). Parents prefer to provide a nurturing environment for adolescents, while early adolescents want to gain more autonomy and independence (74). Adolescents may interpret parenting behavior as hindering their independence, leading to a discrepancy in perceived parenting behavior. Additionally, the measurement of maternal emotion socialization may explain the informant discrepancy. Items like “When I was sad, my mother helped me deal with the issue that made me sad” in the EAC questionnaire are considered emotion coaching. However, if the parent gives advice immediately before validating their child’s emotions in response, the child might perceive the advice as criticism and emotion dismissing, while the parent would perceive it as supportive parental emotion socialization (75).

However, our results did not support the hypothesis that informant discrepancies would be associated with adolescent ER. This finding may support the hypothesis of different agendas, suggesting that the discrepancy arises from normal developmental changes during adolescence and is not reflective of family dysfunction which might negatively impact the adolescent. Although the informant discrepancies in maternal emotion socialization were not associated with adolescent ER, based on the maladaptive hypothesis, they may still have implications for other aspects of adolescent functioning, which could be investigate in further research.

Although our result showed that informant discrepancies in maternal emotion socialization were not related to adolescent ER, we further probed the potential reasons underlying the reported discrepancies. Results did not support De Los Reyes et al. (32)’s notion that parents’ and adolescents’ psychopathological symptoms contribute to the informant discrepancies. In our study, neither mother nor adolescent ER was significantly related to mothers’ and adolescents’ reports of maternal emotion socialization. However, there was a trend for a moderating role of maternal difficulties in ER in the association between mother- and adolescent-reported emotion coaching, such that greater difficulties were associated with greater concordance between informant reports of emotion coaching, which is inconsistent with our hypothesis. Prior research, however, has found that higher levels of parents’ own psychopathology (specifically, depressive) symptoms are related to greater correspondence between different informant ratings of parenting behavior (31). Given that difficulties in ER are known to be related to depression (76), these findings could support the depression realism hypothesis, suggesting that individuals experiencing depression may be more accurate in perceiving or judging their own behavior compared to non-depression individuals (77). It is possible that mothers with greater difficulties in ER may be more aware of their maternal emotion socialization and have more concordance with adolescent reports. Although the moderating role of maternal ER was not statistically significant in the current study, maternal emotion regulation may potentially contribute to the informant discrepancy in maternal emotion socialization.

### 4.1 Limitations and future directions

While this study has several strengths (including use of both self-report and observational data), there are also limitations. First, our sample size was relatively small, so we may not have had sufficient power to detect smaller effects if they existed. Thus, our null findings should be taken with caution and replicated further. Second, our emotion discussion tasks were conducted via video conferencing and the discussion was limited to three emotions with only five minutes per emotion. Further, we did not investigate positive emotions in the discussion task. The video-conferencing environment and time limit may make mothers and adolescents’ behavior less natural and not representative of their daily interactions. This limitation might explain why observed measurements of maternal emotion socialization were not correlated with adolescent ER. Third, we focused on emotion coaching and emotion dismissing broadly, possibly overlooking the nuanced and complex nature of maternal emotion socialization related to specific emotions. Emotion coaching and dismissing also do not represent the entire range of parental emotion socialization, which also includes parents’ beliefs of emotions and parents’ expression of emotion (6). Fourth, our study did not test the interaction hypothesis raised by Gottman et al. (7) that emotion coaching’s main function is to inhibit the negative effect of emotion dismissing. Whether emotion coaching has a moderating role in the association between emotion dismissing and emotional outcomes in adolescents with internalizing symptoms should be examined in the future to test the inhibiting effect of emotion coaching.

Fifth, gender of both parents and their children may influence the effect of parental emotion socialization on adolescent emotional competence (6). Therefore, our findings may not be generalized to fathers and male adolescents. Additionally, cultural factors have been found to influence parental emotion socialization and the majority of participants in the current study were Caucasian and had a high socioeconomic status (78). Thus, our results may not reflect patterns across all families. Furthermore, our sample did not include young people using psychotropic medications, which might limit the generalizability of findings to clinical samples. It will be important to expand this research to samples with different demographic features to gain a more comprehensive understanding of the effect of parental emotion socialization on the development of ER in early adolescents. Sixth, the current study investigated a limited number of factors that may influence informant discrepancies. Discrepant reports have challenged researchers and clinicians in drawing research conclusions and assessing adolescent psychopathology for treatment (20). The role of informant discrepancies in adolescent behavioral reports and their associated features is critical for a comprehensive understanding of adolescent psychopathology. This knowledge could lead to a more accurate measurement of family interaction. Therefore, further studies on the factors associated with informant discrepancy in maternal emotion socialization are needed to provide a deeper understanding of the reasons for the different perceptions of maternal parenting behavior between adolescents and their mothers.

### 4.2 Conclusion and implications

In the present study, results suggest that adolescent-reported maternal emotion socialization is associated with adolescent self-reported ER in female adolescents with elevated internalizing symptoms, and there was a trend suggesting that mothers’ ER might influence their reports of emotion coaching. Although this latter result was not statistically significant, it suggests that when clinicians or researchers measure the outcome of parenting interventions targeting adolescents, it is important to use a multi-informant approach and consider factors that may affect reporting. Further, our findings also suggest that adolescent-reported parenting might be more relevant to adolescents’ ER. It is important to consider how adolescents perceive maternal emotion socialization rather than other’s perspectives. However, further research on the extent of the effect of maternal emotion socialization on ER in adolescents with elevated internalizing symptoms is warranted.

## Data Availability

The datasets presented in this article are not readily available because data cannot be made publicly available in order to protect participant privacy as per the approved ethics requirement. Requests to access the datasets should be directed to swhittle@unimelb.edu.au.
