# Supplementary material for: Association between maternal emotion socialization and emotion regulation in early adolescents with elevated internalizing symptoms: insights from multi-informant discrepancies
Source: Front Psychiatry. 2025 Mar 5;16:1497007. doi: 10.3389/fpsyt.2025.1497007 (PMC11920575; doi:10.3389/fpsyt.2025.1497007)
Supplement: Supplementary file 1 [file DataSheet1.docx]

Supplementary Material

# Supplementary Data

Supplementary analysis examined associations between demographic variables and study variables. The correlation analysis indicated that family composition was significantly associated with mother-reported emotion coaching (*b* = -0.36, *p* < .005) and mother-reported emotion dismissing (*b* = 0.43, *p* < .001). Mothers from two-parent families were more likely to rate higher in emotion dismissing and lower in emotion coaching skills compared to mothers from step-families and sole-parent families. Additionally, mothers’ race was related to mothers’ emotion dysregulation (*b* = -0.31, *p* < .01). Caucasian mothers reported better emotion regulation capacity compared to Asians and Aboriginals.

# Supplementary Tables

**Table S1**. Bivariate Correlations.

|  | EC | ED | PR ED | PR EC | AR ER | PR ER | Mother ER | Fam income | Fam comp. | Mother race | Adol IS | Mother IS | PS | OR EC | OR ED |
| --- | --- | --- | --- | --- | --- | --- | --- | --- | --- | --- | --- | --- | --- | --- | --- |
| EC | 1.00 |  |  |  |  |  |  |  |  |  |  |  |  |  |  |
| ED | -0.71 *** | 1.00 |  |  |  |  |  |  |  |  |  |  |  |  |  |
| PR ED | -0.35 ** | 0.29* | 1.00 |  |  |  |  |  |  |  |  |  |  |  |  |
| PR EC | 0.39 *** | -0.27 * | -0.81 *** | 1.00 |  |  |  |  |  |  |  |  |  |  |  |
| AR ER | -0.25 * | 0.37 ** | 0.02 | 0.05 | 1.00 |  |  |  |  |  |  |  |  |  |  |
| PR ER | -0.04 | 0.13 | 0.20 | -0.16 | 0.23 | 1.00 |  |  |  |  |  |  |  |  |  |
| Mother ER | -0.18 | 0.19 | 0.45 *** | -0.43 *** | -0.09 | 0.25* | 1.00 |  |  |  |  |  |  |  |  |
| Fam income | 0.01 | -0.08 | -0.02 | -0.10 | 0.15 | -0.10 | -0.15 | 1.00 |  |  |  |  |  |  |  |
| Fam comp. | 0.07 | 0.06 | -0.36 ** | 0.43 *** | 0.06 | -0.03 | 0.07 | -0.32 ** | 1.00 |  |  |  |  |  |  |
| Mother race | 0.09 | -0.17 | -0.16 | 0.13 | 0.03 | -0.02 | -0.31 ** | -0.01 | 0.10 | 1.00 |  |  |  |  |  |
| Adol IS | -0.23 | 0.27* | 0.03 | 0.06 | 0.48 *** | 0.12 | -0.06 | 0.03 | 0.17* | 0.09 | 1.00 |  |  |  |  |
| Mother IS | -0.11 | 0.13 | 0.35 ** | -0.25 * | -0.01 | 0.27* | 0.74 *** | -0.10 | 0.08 | -0.21* | 0.01 | 1.00 |  |  |  |
| PS | -0.21 | 0.16 | 0.03 | -0.13 | 0.12 | 0.02 | 0.13 | 0.00 | 0.09 | -0.21* | 0.04 | 0.05 | 1.00 |  |  |
| OR EC | 0.25 *** | -0.22 ** | -0.31 ** | 0.26 *** | -0.17 * | 0.02 | -0.22 ** | -0.07 | 0.10 | 0.10 | -0.17 * | -0.15 | 0.02 | 1.00 |  |
| OR ED | -0.33 *** | 0.26 *** | 0.40 *** | -0.44 *** | 0.12 | 0.02 | 0.22 ** | 0.09 | -0.19 * | 0.03 | 0.05 | 0.18* | 0.06 | -0.83 *** | 1.00 |

*Note*. AR = adolescent-reported, MR = mother-reported, OR = observer-reported, EC = emotion coaching, ED = emotion dismissing, ER = emotion regulation, Mother ER = self-reported maternal emotion regulation, IS = internalizing symptoms, PS = puberty status, Fam = family, comp. = composition, Adol = adolescent. **p*<0.05, ***p*<0.01, ****p*<0.001.

Tables S2 and S3 present results from the polynomial regression analyses with adolescent-reported adolescent emotion regulation as the outcome.

**Table S2.**

Association between discrepancy in mother and adolescent reports of maternal emotion socialization and adolescent-reported emotion regulation.

|  | | Emotion Coaching | | | Emotion Dismissing | | |
| --- | --- | --- | --- | --- | --- | --- | --- |
| Parameter | *β* | | *SE* | *p* | *β* | *SE* | *p* |
| Adol-report | -0.50 | | 0.18 | .008** | 0.38 | 0.14 | .008** |
| Mother-report | 0.19 | | 0.25 | .442 | -0.14 | 0.18 | .446 |
| Adol-report^2^ | -0.02 | | 0.02 | .318 | -1e-03 | 0.01 | .842 |
| Mother-report^2^ | -0.11 | | 0.05 | .022* | -1e-03 | 0.02 | .977 |
| Adol. X Mother | 0.07 | | 0.04 | .136 | -0.01 | 0.02 | .752 |

Note. **p*<0.05, ***p*<0.01. Adol = adolescent; X = interaction; *SE* = standard error

**Table S3.**

Association between discrepancy in observer and adolescent reports of maternal emotion socialization and adolescent-reported emotion regulation.

|  | Emotion Coaching | | | | Emotion Dismissing | | | |  |
| --- | --- | --- | --- | --- | --- | --- | --- | --- | --- |
| Parameter | | *β* | *SE* | *p* | | *β* | *SE* | *p* | |
| Adol-report | | -0.28 | 0.18 | .122 | | 0.34 | 0.13 | .014* | |
| Observer-report | | -1.12 | 1.23 | .368 | | 0.41 | 1.07 | .703 | |
| Adol-report^2^ | | -1e-03 | 0.02 | .948 | | -5.3e-05 | 0.01 | .994 | |
| Observer-report^2^ | | -0.37 | 0.91 | .688 | | -0.26 | 0.85 | .765 | |
| Adol. X observer | | 0.16 | 0.18 | .377 | | -0.06 | 0.12 | .609 | |

Note. **p*<0.05, ***p*<0.01. Adol = adolescent; X = interaction; *SE* = standard error

Tables S4 and S5 present results from the polynomial regression analysis with mother-reported adolescent emotion regulation as outcome.

**Table S4.**

Association between discrepancy in mother and adolescent reports of maternal emotion socialization and mother-reported adolescent emotion regulation.

|  | | Emotion Coaching | | | Emotion Dismissing | | |
| --- | --- | --- | --- | --- | --- | --- | --- |
| Parameter | *β* | | *SE* | *p* | *β* | *SE* | *p* |
| Mother-report | -0.57 | | 0.54 | .300 | 0.29 | 0.38 | .453 |
| Adol-report | 0.47 | | 0.10 | .242 | 0.21 | 0.30 | .484 |
| Mother-report^2^ | 0.08 | | 0.39 | .436 | 0.04 | 0.04 | .256 |
| Adol-report^2^ | 0.09 | | 0.05 | .074 | 1e-03 | 0.01 | .965 |
| Mother X Adol. | -0.02 | | 0.10 | .803 | 0.02 | 0.04 | .584 |

Note. **p*<0.05, ***p*<0.01. Adol = adolescent; X = interaction; *SE* = standard error

**Table S5.**

Association between discrepancy in mother and observer reports of maternal emotion socialization and mother-reported adolescent emotion regulation.

|  | Emotion Coaching | | | | Emotion Dismissing | | | |  |
| --- | --- | --- | --- | --- | --- | --- | --- | --- | --- |
| Parameter | | *β* | *SE* | *p* | | *β* | *SE* | *p* | |
| Mother-report | | -0.55 | 0.53 | .303 | | 0.47 | 0.35 | .726 | |
| Observer-report | | 1.58 | 2.61 | .548 | | -2.28 | 2.37 | .470 | |
| Mother-report^2^ | | 0.02 | 0.09 | .812 | | 0.02 | 0.03 | .340 | |
| Observer-report^2^ | | -0.47 | 1.91 | .804 | | -1.43 | 2.11 | .501 | |
| Mother X observer | | 0.46 | 0.52 | .379 | | 0.43 | 0.32 | .175 | |

Note. **p*<0.05, ***p*<0.01. Adol = adolescent; X = interaction; *SE* = standard error

**3 Supplementary analyses**

None of the interaction terms involving adolescent report (adolescent-report X mother-report emotion socialization; adolescent-report X observer-report emotion socialization) reached statistical significance, indicating that the discrepancies between adolescent and other reports of emotion socialization were not associated with adolescent internalizing symptoms, maternal internalizing symptoms, family composition, and mother’s race (Table S6-S14).

Tables S6 and S7 present results from the polynomial regression analyses with adolescent internalizing symptoms as the outcome.

**Table S6.**

Association between discrepancy in mother and adolescent reports of maternal emotion socialization and adolescent internalizing symptoms.

|  | | Emotion Coaching | | | Emotion Dismissing | | |
| --- | --- | --- | --- | --- | --- | --- | --- |
| Parameter | *β* | | *SE* | *p* | *β* | *SE* | *p* |
| Adol-report | -0.52 | | 0.34 | .126 | 0.46 | 0.26 | .071 |
| Mother-report | 0.63 | | 0.46 | .181 | -0.05 | 0.33 | .883 |
| Adol-report^2^ | 0.04 | | 0.04 | .398 | -4.3e-03 | 0.01 | .716 |
| Mother-report^2^ | -0.01 | | 0.08 | .725 | -8.7e-03 | 0.03 | .777 |
| Adol. X Mother | 0.03 | | 0.08 | .725 | 0.02 | 0.03 | .531 |

Note. **p*<0.05, ***p*<0.01. Adol = adolescent; X = interaction; *SE* = standard error

**Table S7.**

Association between discrepancy in observer and adolescent reports of maternal emotion socialization and adolescent internalizing symptoms.

|  | Emotion Coaching | | | | Emotion Dismissing | | | |  |
| --- | --- | --- | --- | --- | --- | --- | --- | --- | --- |
| Parameter | | *β* | *SE* | *p* | | *β* | *SE* | *p* | |
| Adol-report | | -0.30 | 0.31 | .357 | | 0.50 | 0.25 | .046* | |
| Observer-report | | -1.74 | 2.18 | .429 | | -0.77 | 1.96 | .697 | |
| Adol-report^2^ | | 0.04 | 0.04 | .287 | | -0.01 | 0.01 | .538 | |
| Observer-report^2^ | | -1.09 | 1.62 | .503 | | -0.53 | 1.57 | .735 | |
| Adol. X observer | | 0.38 | 0.32 | .231 | | 0.30 | 0.22 | .178 | |

Note. **p*<0.05, ***p*<0.01. Adol = adolescent; X = interaction; *SE* = standard error

Tables S8 and S9 present results from the polynomial regression analyses with family composition as the outcome.

**Table S8.**

Association between discrepancy in mother and observer reports of maternal emotion socialization and family composition.

|  | Emotion Coaching | | | | Emotion Dismissing | | | |
| --- | --- | --- | --- | --- | --- | --- | --- | --- |
| Parameter | | *β* | *SE* | *p* | | *β* | *SE* | *p* |
| Mother-report | | 0.10 | 0.02 | <.001*** | | -0.06 | 0.02 | <.001 |
| Adol-report | | 3.6e-03 | 0.02 | .833 | | 0.03 | 1.8e-03 | .101 |
| Mother-report^2^ | | 0.01 | 4.3e-03 | .016* | | 2.3e-03 | 1.6e-03 | .156 |
| Adol-report^2^ | | 4.1e-03 | 2.2e-03 | .065 | | 3.3e-05 | 6.2e-04 | .957 |
| Mother X Adol. | | -6.3e-03 | 4.0e-03 | .122 | | -2.6e-03 | 1.8e-03 | .148 |

Note. **p*<0.05, ***p*<0.01. Adol = adolescent; X = interaction; *SE* = standard error

**Table S9.**

Association between discrepancy in mother and observer reports of maternal emotion socialization and family composition.

|  | Emotion Coaching | | | | Emotion Dismissing | | | |  |
| --- | --- | --- | --- | --- | --- | --- | --- | --- | --- |
| Parameter | | *β* | *SE* | *p* | | *β* | *SE* | *p* | |
| Mother-report | | 0.10 | 0.02 | <.001 | | -0.04 | 0.02 | .010* | |
| Observer-report | | -0.01 | 0.11 | .957 | | -0.07 | 0.11 | .551 | |
| Mother-report^2^ | | 0.01 | 3.8e-03 | .149 | | 3.6e-04 | 1.6e-03 | .816 | |
| Observer-report^2^ | | 0.08 | 0.08 | .328 | | 0.04 | 0.10 | .716 | |
| Mother X observer | | 0.01 | 0.02 | .804 | | 5.1e-03 | 0.15 | .730 | |

Note. **p*<0.05, ***p*<0.01. Adol = adolescent; X = interaction; *SE* = standard error

Tables S10 and S11 present results from the polynomial regression analyses with mother’s race as the outcome.

**Table S10.**

Association between discrepancy in mother and observer reports of maternal emotion socialization and mother’s race.

|  | Emotion Coaching | | | | Emotion Dismissing | | | |
| --- | --- | --- | --- | --- | --- | --- | --- | --- |
| Parameter | | *β* | *SE* | *p* | | *β* | *SE* | *p* |
| Mother-report | | 0.02 | 0.03 | .572 | | -6.1e-03 | 0.02 | .787 |
| Adol-report | | 0.01 | 0.02 | .584 | | -0.02 | 0.02 | .190 |
| Mother-report^2^ | | 3.6e-03 | 6.0e-03 | .544 | | -1.6e-03 | 2.1e-03 | .435 |
| Adol-report^2^ | | 1.9e-03 | 3.0e-03 | .522 | | 3.9e-04 | 8.1e-04 | .635 |
| Mother X Adol. | | -7.9e-03 | 5.6e-03 | .162 | | 8.1e-04 | 2.3e-03 | .728 |

Note. **p*<0.05, ***p*<0.01. Adol = adolescent; X = interaction; *SE* = standard error

**Table S11.**

Association between discrepancy in mother and observer reports of maternal emotion socialization and mother’s race.

|  | Emotion Coaching | | | | Emotion Dismissing | | | |  |
| --- | --- | --- | --- | --- | --- | --- | --- | --- | --- |
| Parameter | | *β* | *SE* | *P* | | *β* | *SE* | *p* | |
| Mother-report | | 0.02 | 0.03 | .514 | | -0.02 | 0.02 | .308 | |
| Observer-report | | 0.10 | 0.15 | .526 | | 0.10 | 0.14 | .469 | |
| Mother-report^2^ | | -5.6e-04 | 0.01 | .914 | | -1.3e-03 | 2.0e-03 | .505 | |
| Observer-report^2^ | | -0.05 | 0.11 | .659 | | -0.14 | 0.12 | .256 | |
| Mother X observer | | -0.01 | 0.03 | .838 | | 0.02 | 0.02 | .291 | |

Note. **p*<0.05, ***p*<0.01. Adol = adolescent; X = interaction; *SE* = standard error

Tables S12 and S13 present results from the polynomial regression analyses with maternal internalizing symptoms as the outcome.

**Table S12.**

Association between discrepancy in mother and observer reports of maternal emotion socialization and maternal internalizing symptoms.

|  | Emotion Coaching | | | | Emotion Dismissing | | | |
| --- | --- | --- | --- | --- | --- | --- | --- | --- |
| Parameter | | *β* | *SE* | *p* | | *β* | *SE* | *p* |
| Mother-report | | -5.89 | 2.64 | .029* | | -2.68 | 0.88 | .003** |
| Adol-report | | 0.30 | 1.43 | .836 | | 0.75 | 0.63 | .243 |
| Mother-report^2^ | | 0.06 | 0.04 | .148 | | 0.05 | 0.01 | <.001*** |
| Adol-report^2^ | | -0.02 | 0.02 | .420 | | -0.01 | 0.01 | .332 |
| Mother X Adol. | | 0.02 | 0.04 | .569 | | -0.01 | 0.02 | .720 |

Note. **p*<0.05, ***p*<0.01. Adol = adolescent; X = interaction; *SE* = standard error

**Table S13.**

Association between discrepancy in mother and observer reports of maternal emotion socialization and maternal internalizing symptoms.

|  | Emotion Coaching | | | | Emotion Dismissing | | | |  |
| --- | --- | --- | --- | --- | --- | --- | --- | --- | --- |
| Parameter | | *β* | *SE* | *P* | | *β* | *SE* | *p* | |
| Mother-report | | -5.22 | 2.53 | .043* | | -2.25 | 0.82 | .008** | |
| Observer-report | | -16.95 | 8.42 | .048* | | -7.75 | 4.29 | .076 | |
| Mother-report^2^ | | 0.06 | 0.77 | .129 | | 0.03 | 0.01 | .014* | |
| Observer-report^2^ | | 0.86 | 0.04 | .271 | | 0.56 | 0.83 | .503 | |
| Mother X observer | | 0.29 | 0.21 | .176 | | 0.14 | 0.12 | .256 | |

Note. **p*<0.05, ***p*<0.01. Adol = adolescent; X = interaction; *SE* = standard error

To investigate the contribution of both adolescent and maternal internalizing symptoms, family composition, and mother’s race to the informant discrepancy for emotion socialization. Linear regression models revealed that neither adolescent nor maternal internalizing symptoms significantly moderated the associations between maternal emotion socialization reported by different informants (Table S14,S17). Similarly, neither family composition nor mother’s race significantly moderated the associations between maternal emotion socialization reported by different informants (Table S15, S16).

**Table S14.**

Linear regression testing the contribution of adolescent internalizing symptoms to informant discrepancies.

|  | Dependent variable: Adolescent-reported emotion coaching | | | | |
| --- | --- | --- | --- | --- | --- |
|  | | *β* | *SE* | *p* | R^2^ |
| Adolescent IS | | -0.10 | 0.04 | .046* | .28 |
| Mother-reported emotion coaching | | 0.57 | 0.17 | .001** |  |
| Observed emotion coaching | | 1.05 | 0.87 | .231 |  |
| Mother-reported x Adolescent IS | | 4.2e-03 | 9.2e-03 | .650 |  |
| Observed x Adolescent IS | | 0.12 | 0.07 | .081 |  |

|  | Dependent variable: Adolescent-reported emotion dismissing | | | | |
| --- | --- | --- | --- | --- | --- |
|  | | *β* | *SE* | *p* | R^2^ |
| Adolescent IS | | 0.17 | 0.07 | .023** | .19 |
| Mother-reported emotion dismissing | | 0.27 | 0.16 | .116 |  |
| Observed emotion dismissing | | 1.52 | 1.19 | .205 |  |
| Mother-reported x Adolescent IS | | -2.2e-03 | 0.01 | .818 |  |
| Observed x Adolescent IS | | 0.09 | 0.08 | .258 |  |

Note. **p*<0.05, ***p*<0.01. IS = internalizing symptoms, *SE* = standard error

**Table S15.**

Linear regression testing the contribution of family composition to informant discrepancies.

|  | Dependent variable: Adolescent-reported emotion coaching | | | | |
| --- | --- | --- | --- | --- | --- |
|  | | *β* | *SE* | *p* | R^2^ |
| Adolescent FC | | -0.90 | 1.30 | .489 | .21 |
| Mother-reported emotion coaching | | 0.59 | 0.20 | .005** |  |
| Observed emotion coaching | | 1.36 | 0.90 | .135 |  |
| Mother-reported x Adolescent FC | | 0.01 | 0.26 | .955 |  |
| Observed x Adolescent FC | | -1.10 | 0.83 | .191 |  |

|  | Dependent variable: Adolescent-reported emotion dismissing | | | | |
| --- | --- | --- | --- | --- | --- |
|  | | *β* | *SE* | *p* | R^2^ |
| Adolescent FC | | 3.82 | 1.68 | .036* | .17 |
| Mother-reported emotion dismissing | | 0.48 | 0.19 | .013* |  |
| Observed emotion dismissing | | 1.70 | 1.19 | .157 |  |
| Mother-reported x Adolescent FC | | 0.29 | 0.24 | .237 |  |
| Observed x Adolescent FC | | 0.66 | 1.27 | .606 |  |

*Note*. **p*<0.05, ***p*<0.01, ****p*<0.001. FC = family composition, *SE* = standard error

**Table S16.**

Linear regression testing the contribution of mother’s race to informant discrepancies.

|  | Dependent variable: Mother-reported emotion coaching | | | | |  |
| --- | --- | --- | --- | --- | --- | --- |
|  | | *β* | *SE* | *p* | R^2^ | |
| Mother race | | 0.21 | 0.52 | .689 | .21 | |
| Adolescent-reported emotion coaching | | 0.22 | 0.08 | .005** |  | |
| Observed emotion coaching | | 0.98 | 0.59 | .103 |  | |
| Adolescent reported x Mother race | | -0.08 | 0.07 | .242 |  | |
| Observed x Mother race | | -0.05 | 0.50 | .921 |  | |

|  |  | Dependent variable: Mother-reported emotion dismissing | | | | |
| --- | --- | --- | --- | --- | --- | --- |
|  | | | *β* | *SE* | *p* | R^2^ |
| Mother race | | | -1.12 | 0.84 | .186 | .22 |
| Adolescent-reported emotion dismissing | | | 0.13 | 0.09 | .153 |  |
| Observed emotion dismissing | | | 2.59 | 0.83 | .003** |  |
| Adolescent- reported x Mother race | | | 0.03 | 0.08 | .721 |  |
| Observed x Mother race | | | 0.22 | 0.60 | .717 |  |

*Note*. **p*<0.05, ***p*<0.01, ****p*<0.001. *SE* = standard error

**Table S17.**

Linear regression testing the contribution of maternal internalizing symptoms to informant discrepancies.

|  | Dependent variable: Mother-reported emotion coaching | | | | |  |
| --- | --- | --- | --- | --- | --- | --- |
|  | | *β* | *SE* | *p* | R^2^ | |
| Mother IS | | 1.6e-03 | 0.64 | .998 | .18 | |
| Adolescent-reported emotion coaching | | 0.49 | 0.35 | .167 |  | |
| Observed emotion coaching | | 1.82 | 1.78 | .309 |  | |
| Adolescent reported x Mother IS | | 2.6e-03 | 0.02 | .887 |  | |
| Observed x Mother IS | | -0.04 | 0.10 | .705 |  | |

|  |  | Dependent variable: Mother-reported emotion dismissing | | | | |
| --- | --- | --- | --- | --- | --- | --- |
|  | | | *β* | *SE* | *p* | R^2^ |
| Mother IS | | | 0.79 | 0.55 | .156 | .14 |
| Adolescent-reported emotion dismissing | | | 0.72 | 0.34 | .040* |  |
| Observed emotion dismissing | | | 1.32 | 2.34 | .575 |  |
| Adolescent- reported x Mother IS | | | -0.03 | 0.02 | .159 |  |
| Observed x Mother IS | | | 0.03 | 0.13 | .789 |  |

*Note*. **p*<0.05, ***p*<0.01, ****p*<0.001. *IS* = internalizing symptoms, *SE* = standard error
